# Assessing a Couples-Based, Digital HIV Serostatus-Neutral Intervention (Para Ti, Para Mí, Para Nosotros) for Adult Cisgender Sexual Minority Male Couples in Lima, Peru: Protocol for a 6-Month Pilot Randomized Controlled Trial

**DOI:** 10.2196/63106

**Published:** 2024-10-10

**Authors:** Jason W Mitchell, Zoran Bursac, David Diaz, Edward Michael Reyes Diaz, Alfonso Silva-Santisteban, Kelika A Konda

**Affiliations:** 1 Department of Health Promotion and Disease Prevention Robert Stempel College of Public Health and Social Work Florida International University Miami, FL United States; 2 Department of Biostatistics Robert Stempel College of Public Health and Social Work Florida International University Miami, FL United States; 3 Centro de Investigación Interdisciplinaria en Sexualidad, SIDA y Sociedad Universidad Peruana Cayetano Heredia Lima Peru; 4 Department of Population and Public Health Sciences Keck School of Medicine University of Southern California Los Angeles, CA United States

**Keywords:** digital health intervention, HIV prevention care, sexually transmitted infection, cisgender, sexual minority male couples, randomized controlled trial, Peru, mobile phone

## Abstract

**Background:**

HIV disproportionately affects sexual minority men (SMM; eg, gay, bisexual, and other men who have sex with men) in Lima, Peru; epidemiological data estimate that 32% to 39% of new HIV infections occur among adult cisgender SMM within primary partnerships (ie, male couples). Most HIV prevention-care research in Lima, Peru, has focused on SMM as individuals and not couples. To help address this critical gap in prevention care, we developed Para Ti, Para Mí, Para Nosotros (P3): a couples-based, digital HIV serostatus-neutral intervention (DHI) for adult cisgender SMM couples in Lima, Peru. The P3 DHI is designed to facilitate couples with skill-building, communication, decision-making, and working together to form and adhere to a detailed prevention care plan that aligns with their explicit sexual agreement. The P3 DHI is theoretically informed, self-guided, directed, sequential, and fully automated.

**Objective:**

This pilot randomized controlled trial (RCT) aims to examine the preliminary effects of P3 on couples’ formation and adherence to a detailed prevention care plan containing evidence-based strategies that also aligns with their explicit sexual agreement over time. In addition, the feasibility of enrollment and retention and couples’ acceptability of P3 will be assessed.

**Methods:**

The research implements a prospective, 6-month pilot RCT with a 3-month delayed control condition. After baseline, 60 enrolled SMM couples will be randomized to 1 of 2 conditions. Couples randomized to the unmatched, delayed control condition will receive access to the P3 DHI to use during the last 3 months of the trial after the 3-month assessment. Couples randomized to the immediate intervention condition will immediately receive access to the P3 DHI for 6 months. Study assessments will occur at baseline and months 3 and 6. Descriptive, comparative, qualitative, and longitudinal analyses using generalized linear mixed-effect, multilevel, and actor-partner interdependence models will be conducted to address the specific aims.

**Results:**

The 6-month pilot RCT is ongoing. Recruitment, enrollment, and data collection began in January 2023 and ended in April 2024. A total of 74 adult cisgender SMM couples met all inclusion criteria, provided consent, and were enrolled in the pilot RCT. Retention was 92% (68/74) at month 6. Data are currently being analyzed to address the 3 specific aims regarding feasibility, acceptability, and preliminary efficacy.

**Conclusions:**

Findings from this research will reveal whether couples deemed the P3 DHI to be acceptable. Findings will also highlight the preliminary efficacy of the P3 DHI on couples managing their vulnerability to HIV and other sexually transmitted infections (STIs) over time via alignment of their prevention-care plan and sexual agreement. Trial findings will help shape the future direction of the P3 DHI while addressing the existing gap in prevention and care services for couples in the local context.

**Trial Registration:**

ClinicalTrials.gov NCT05873855; https://clinicaltrials.gov/study/NCT05873855

**International Registered Report Identifier (IRRID):**

DERR1-10.2196/63106

## Introduction

### Background

Peru, similar to much of Latin America, has a concentrated HIV epidemic, where cisgender, sexual minority men (SMM; ie, gay, bisexual, and other men who have sex with men) are the primary population affected by HIV. While estimates vary across studies, most studies estimate 10% of HIV prevalence [1,2]; recent incidence estimates continue to be high, estimating an incidence rate of >6 cases per 100 person-years [3]. While there have been fewer studies focusing on sexually transmitted infections (STIs), existing studies point to a similar epidemic pattern [4,5]. Furthermore, epidemiological estimates indicate that between 32% and 39% of new HIV infections among adult cisgender SMM occur within primary partnerships (ie, among male couples) [6].

Although couples have been considered an important population for HIV prevention, no interventions focusing on couples have been tested or implemented in Peru. In this context, same-sex couples (eg, sexual and gender minorities) remain unacknowledged by Peruvian law and exist within a context of limited social and societal support [7]. In addition, while the health system provides both antiretroviral therapy (ART) and pre-exposure prophylaxis (PrEP), couples have not been integrated into considerations of treatment as prevention or PrEP provision, apart from pregnant women with a partner who is living with HIV. In short, HIV prevention care efforts in Peru have exclusively been limited to individual-level measures.

To attain the goal of expanding services beyond the individual level, we conducted pilot work to better understand the needs and interests of cisgender SMM couples in dyadic approaches to HIV or STI prevention care. Our pilot work included an acceptability project with 42 adult cisgender SMM couples who used a former couples-based, eHealth HIV and STI prevention intervention [8] and provided feedback about their experience of using it and how they would like to have it adapted and improved to best meet their relationship and prevention care needs. Findings from this pilot work have previously been described in detail [9-11]. These findings highlighted that the couples’ acceptability of the intervention was high. In addition, couples’ recommendations to improve the intervention centered on three main areas: (1) revamping it to be serostatus neutral, (2) ensuring that it addresses acceptance and ways to improve relations with their own and partner’s family as well as ways to strengthen the relationship with their partner, and 3) ensuring that it is accessible for use on different web-connected devices to increase reach and availability. Documented evidence from the literature [6] and the aforementioned pilot work represented the completion of the assessment and decision stages of the ADAPT-ITT (Assessment, Decision, Adaptation, Production, Topical Experts, Integration, Training, and Testing) model [12] that led to the decision to adapt the former couples-based eHealth HIV and STI prevention intervention. The remaining stages of the ADAPT-ITT model were followed to adapt the intervention into its current form, which is a couples-based, digital HIV serostatus neutral intervention (DHI) called Para Ti, Para Mí, Para Nosotros (P3).

This paper describes the protocol for a 6-month pilot randomized controlled trial (RCT) of the P3 DHI for adult cisgender SMM couples in Lima, Peru. The P3 DHI is designed to facilitate and enhance skills-building of communication and decision-making as well as to encourage relationship partners to work together toward their shared health and relationship goals, with an emphasis on sexual health, HIV and STI prevention care, and relationship dynamics.

### P3 Structure and Organization

The P3 DHI is a secure web app compliant with the Health Insurance Portability and Accountability Act. It can be used across a variety of different web-connected devices, ranging from smartphones to desktop computers. Two types of end users may access and use the P3 DHI: (1) both members of the couple and (2) administrators such as researchers and organizational staff. All users must create an account to access and use nonpublic components of the P3 DHI web app (eg, intervention and administrative portal). The publicly available and accessible components of the P3 DHI include information about the study trial, access to the eligibility screener, e-consent, contact information verification process, and partner study invite system.

The P3 DHI hosts the couples-based, HIV serostatus–neutral intervention that can be hidden or activated, depending on the study design of the trial and randomization assignment of the couple. The P3 DHI also provides participants with access to assessments that automatically populate over time (ie, closer to the due date) and link directly with Qualtrics (Qualtrics International Inc). Furthermore, the P3 DHI incorporates and uses an automated message reminder system. Participants receive up to 5 different types of SMS text messages. One type of SMS text message is instructional, such as for verifying contact information, for creating a study account to access the DHI, and other similar tasks. Another type of SMS text message serves as a reminder for participants to complete their assessments by a certain due date. An additional SMS text message type provides prompts for partners of the couple to start the intervention, whereas another type of SMS text message provides prompts of encouragement to finish the intervention once started. The final type of SMS text message serves the purpose of informing 1 partner that the other partner has caught up to them in the module. All SMS text message types are automated and sent based on predetermined dates or using actions and inactions.

### Theoretical Foundation of P3

**Figure 1:**
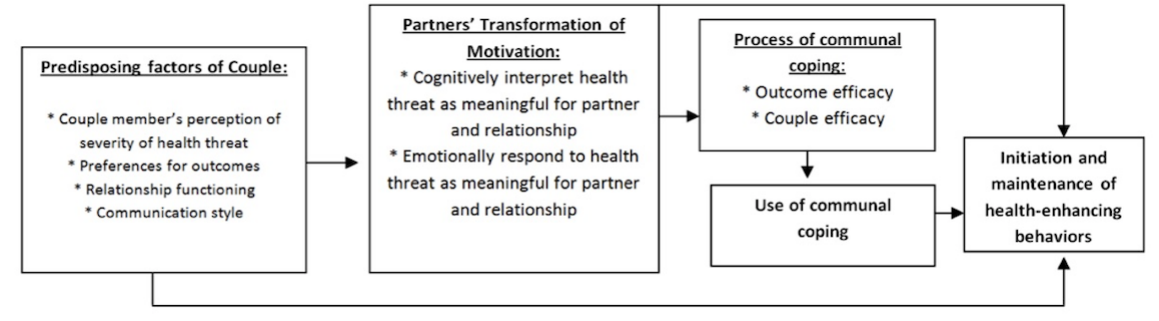
Conceptual model of Couples Interdependence Theory for Health Behavior Change (adapted from Lewis et al [13]).

The P3 DHI is theoretically guided by the Couples Interdependence Theory for Health Behavior Change (CIT-HBC; Figure 1) [13,14]. CIT-HBC accounts for the potential ways partners interact and influence their own and one another’s outcomes and behaviors within the context of their relationship. CIT-HBC provides a useful framework to better understand and encourage health behavior change while considering both partners’ needs and the needs of their relationship (ie, following a couple-based approach). The aim of CIT-HBC is to encourage both the partners to initiate and maintain behaviors that enhance their health (eg, their uptake of and adherence to using evidence-based HIV and STI prevention care strategies over time). CIT-HBC considers how communal coping and transformation of motivation affect the outcomes and behaviors among couples to inform health behavior change. Transformation of motivation accounts for changes in partners’ behavior from a primarily individual-focused motivation to one that is more prorelationship, considering the health needs of both partners and their health-enhancing behaviors [13,14], which is represented via a shift from an individualistic viewpoint to a more shared view of we. Communal coping refers to partners holding a shared assessment of a health threat and a vision of shared action about managing their behaviors and related efforts [13,14] and what both partners will do together to manage their HIV or STI vulnerability. Couple’s interdependence and communal coping approaches have previously been used to understand how health behavior change occurs, including health-enhancing and health-compromising behaviors related to HIV among adult cisgender SMM couples. For example, CIT-HBC is the foundation for couples HIV testing and counseling [15,16]. Regarding measurement for initiating and sustaining health behavior change, CIT-HBC accounts for how partners interact to influence one another’s outcomes through the actor-partner interdependence model (APIM) [17]. APIM permits measurement of actor effects (ie, how a partner influences his own behavior) and partner effects (ie, how each member of the couple influences his partner’s behaviors, independent of their own behaviors).

### P3 Intervention

The couples-based intervention within the P3 DHI is organized into 5 modules. Each module focuses on a core topic with relevant content and related activities, including quizzes, videos, and infographics. Module 1 focuses on communication and decision-making. Module 2 is about HIV and STI prevention care and sexual pleasure. Module 3 centers on sexual agreements. Module 4 is about relationship strengthening, social support, and goal setting. Module 5 focuses on physical activity. All modules reinforce relevancy to sexual health and prevention care. A variety of behavioral change techniques [18] are embedded within each module, including goal setting (behavior), problem solving, action planning, review of behavioral goal, social support (unspecific), social support (practical), information about health consequences, demonstration of the behavior through videos and credible sources, and tailoring and personalization. Drawing from our formative work that led to this pilot RCT protocol, this research refined a previously developed behavioral change technique called dyadic comparison. Dyadic comparison reveals similarities and differences between couple partners to help further their understanding about sexual health and related topics by facilitating communication, decision-making, and goal setting for themselves and their relationship.

### P3 User Interface and User Experience

**Figure 2:**
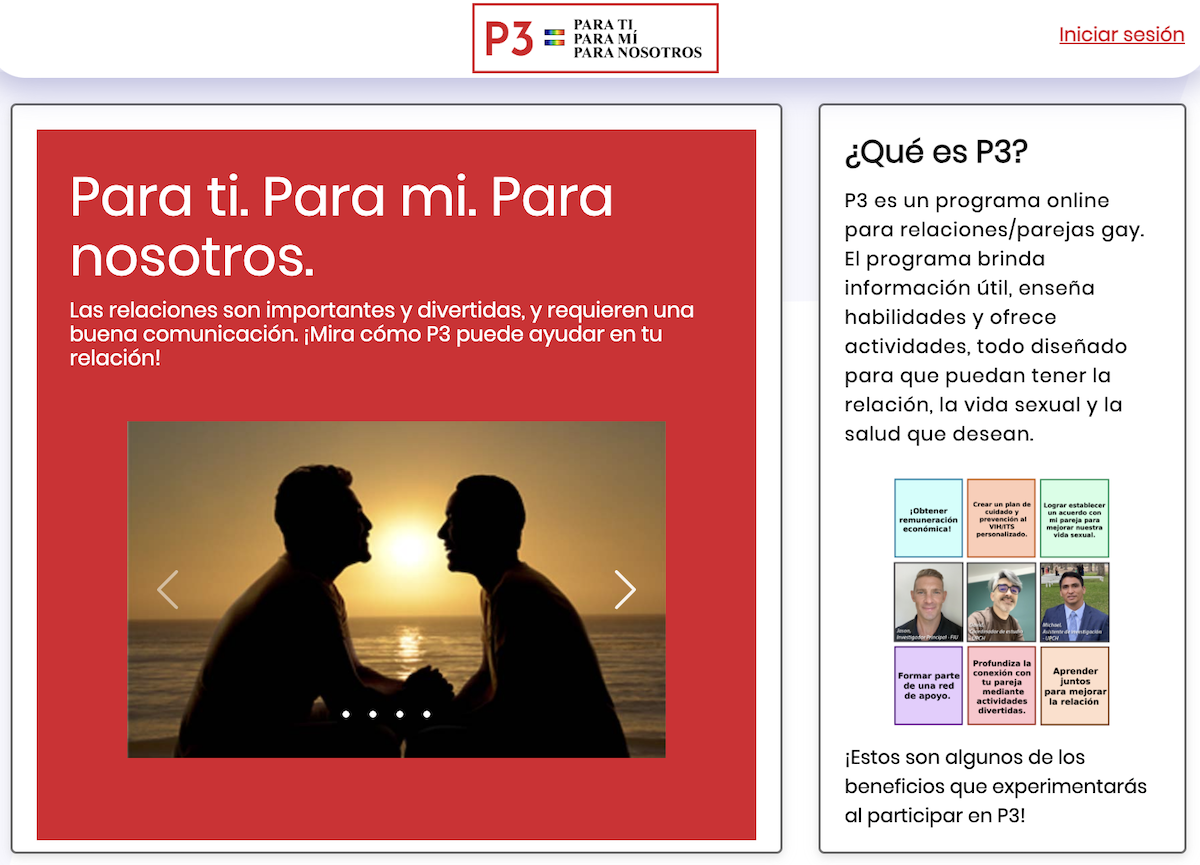
Para Ti, Para Mí, Para Nosotros (P3) web app landing page to access the eligibility screener and the intervention via an “iniciar session.”.

The participant or client user interface of the P3 DHI, that is, what partners and couples experience while using the program, is directed, sequential, and fully automated (ie, without human involvement). Specifically, both the partners must complete module 1 before they can proceed to module 2; they must complete module 2 before accessing module 3, and so forth. Once a module is complete, both the partners may revisit any section of it, including reviewing their responses to the activities and retaking the module. Each module is also organized into subsections that are referred to individually and as a couple, denoted by symbols with instructional text to inform and remind the user of who is involved in each subsection. Estimated time approximations to complete a module and its subsections are also provided at the start of each module (eg, part A: 5 minutes). An estimated time of 15 to 20 minutes is needed to complete a module, excluding any conversation time that would occur between the partners. Figure 2 provides an example screenshot of the P3 DHI.

In addition to a secure SMS text messaging system to communicate with participants, the P3 DHI also contains an administrative and clinical portal for researchers and staff. The administrative portal serves as a one-stop shop to store, organize, and monitor data associated with the trial. Along with its other features, the portal contains a dashboard and participant tracking system. The dashboard provides a global overview of all study participants and couples’ engagement with the study, ranging from data associated with eligibility and enrollment rates and information on assessment and module completions. The dashboard also offers researchers and staff the ability to download data about participants’ and couples’ experiences with regard to using the intervention modules within the P3 DHI. Finally, as each couple progresses through the trial, the participant tracking system provides descriptive details including due and completion dates of assessments as well as when and which modules are completed.

**Figure 3:**
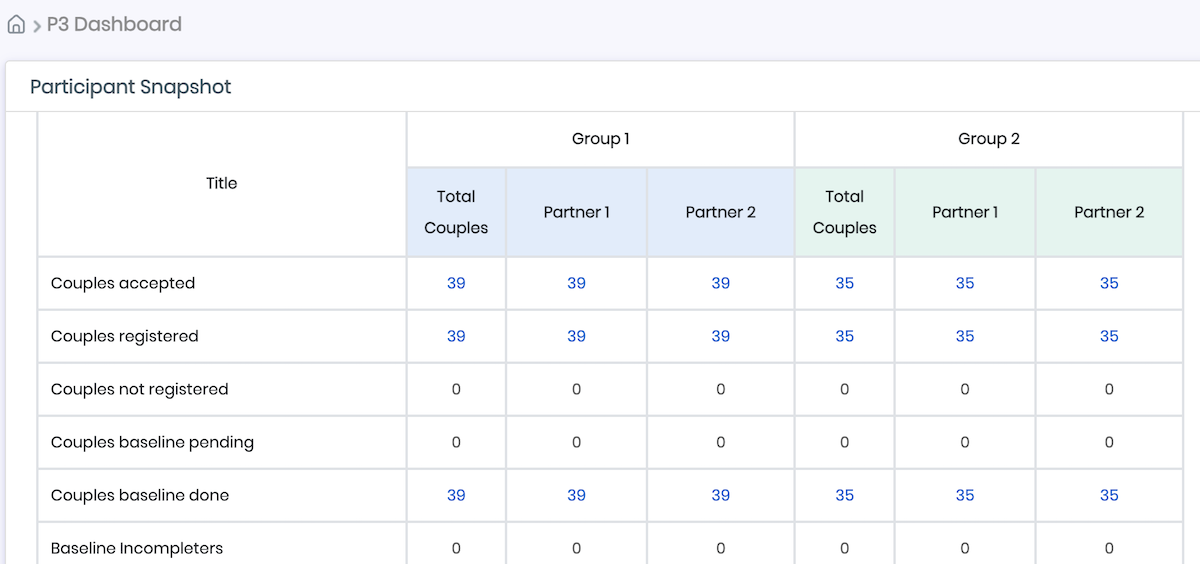
The participant snapshot within the dashboard of the Para Ti, Para Mí, Para Nosotros (P3) administrative portal that provides the number of couples who have met all eligibility criteria (ie, accepted), registered by creating a study profile, and completed the baseline assessment for enrollment.

**Figure 4:**
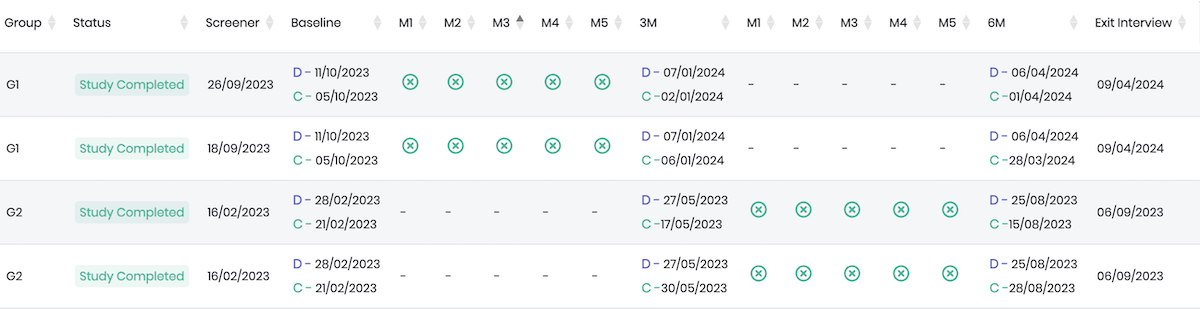
A screenshot of the participant tracking within the Para Ti, Para Mí, Para Nosotros (P3) administrative portal showing the progress of each participant during the pilot randomized controlled trial.

Both the dashboard and participant tracking system are interactive such that all numbers displayed in the dashboard provide the administrative user the ability to learn more about which specific participants or couples are linked to a particular characteristic in the dashboard (eg, number of enrolled couples in the immediate intervention arm). In addition, each participant and couple are assigned a unique ID. Selecting and clicking on the unique ID in the administrative portal leads to a web page providing information about the couple, their assessments (eg, due date and completion date), their module use (date of completion relative to assessment timeline for trial), links to their screener responses, when and which SMS text message reminders they have received, contact information, and HIV and STI testing details, including results. Figures 3 and 4 provide screenshot examples of the administrative portal.

Our formative work with the population, including the use of a community advisory board and human-centered design methods [19-22], informed the overall structure, organizational layout, and user interface and user experience of the alpha and current beta version of the P3 DHI. The beta version of the P3 DHI will be evaluated using a 6-month long pilot RCT.

### Specific Aims

There are 3 specific aims for this pilot RCT with adult cisgender SMM couples in Lima, Peru:

- To assess the feasibility of retaining 60 eligible couples who consented to participate in a 6-month pilot RCT, through quantitative measurement of enrollment, retention, and attrition rates plus reason(s) for attrition.
- To describe the acceptability of P3 using mixed methods [23] from 3 data sources: at final follow-up, the partners’ responses to the Health Information Usability Evaluation Scale [24] and other survey items (eg, 3 things liked best or least about P3 and features for improvement) will be captured, along with the additional details they will share about their experiences during the individual-level, qualitative exit interview. Partners’ paradata will also be descriptively analyzed to describe the patterns of use, such as completion rates, time, and reuse.
- To examine the preliminary efficacy of the P3 DHI on couples through the following measures:
  - Formation and adherence to an HIV and STI prevention care plan containing evidence-based strategies (eg, using ART for undetectable=untransmissible [U=U], PrEP, and routine testing)
  - Formation and adherence to a detailed sexual agreement
  - Relationship functioning (eg, mutual constructive communication, self-efficacy of decision-making, and working together)
  - Self-reported and biomarker-confirmed indicators of vulnerability vis-à-vis sexual behaviors (eg, condomless anal sex [CAS]) and HIV and STIs

To accomplish the above specific aims, data will be collected from both the partners of the couple during the trial; analytic comparisons will be made between the 2 intervention trial conditions. The purpose of this paper is to describe the protocol that will be used to conduct a 6-month pilot RCT of the P3 DHI with adult cisgender SMM couples in Lima, Peru.

## Methods

### Trial Study Design

The study design for the P3 DHI is a 6-month pilot RCT with an unmatched delayed control condition of 3 months. The pilot RCT for the P3 DHI is registered on ClinicalTrials.gov (NCT05873855). Assessments will occur every 3 months after the baseline assessment (at 3 and 6 months). Participants (ie, couples) will initially be blinded to which randomized condition they receive for the pilot RCT. Most study participation activities, including recruitment, eligibility screening, consent, enrollment, assessments, intervention, and exit interviews will occur on Zoom (Zoom Video Communications). During the pilot trial, participants will also undergo in-person HIV and STI testing at a local community-based organization at baseline and month 6. The planned sample size for enrollment is 60 adult cisgender SMM couples. The planned sample size will be balanced by dyad HIV serostatus and stratified by trial arm (ie, 10 seroconcordant negative, 10 serodiscordant, and 10 seroconcordant positive couples per arm). Florida International University (FIU) and Universidad Peruana Cayetano Heredia’s respective institutional review boards have approved the study protocol and all associated study activities.

### Recruitment

Active and passive recruitment strategies will be used to recruit the sample. These strategies include (1) placement of targeted advertisements on social media platforms (eg, Facebook and Instagram [Meta Platforms, Inc], Scuff [Perry Street Software, Inc], and Grindr [Grindr, Inc]); (2) emailing informational flyers to sexual and gender minority communities and HIV and STI service organizations; and (3) referrals from health providers, peers, or relationship partners. All interested participants will be referred to the project website to learn more about the study, complete the brief eligibility screener, provide e-consent, and submit their own and their partners’ contact information (WhatsApp number). Both partners of each couple must complete the eligibility screener, provide e-consent, and enter accurate and complete contact information to be considered for eligibility.

### Inclusion and Exclusion Criteria

#### Overview

This pilot RCT study will use a 3-level approach to determine eligibility. Level 1 will determine an individual’s eligibility based on their responses to the screener (ie, individual level). Level 2 will determine a couple’s eligibility using data from both the participants who indicate that they are in a relationship with one another (ie, couple level). Embedded electronic algorithms within the eligibility screener will automatically determine whether individuals meet the criteria for level 1 eligibility and whether couples meet the criteria for level 2 eligibility, provided that both partners complete the screener, consent, and all contact information. Level 3 will only occur if the inclusion criteria for levels 1 and 2 are met. Unlike the previous levels, the study staff will apply level 3 eligibility criteria during a Zoom study onboarding meeting by asking both partners of the couple to verify some of their responses to the eligibility screener.

#### Level 1: Individual Eligibility

Each individual participant within an adult cisgender SMM couple must meet the following individual-level eligibility criteria for the P3 pilot RCT: (1) currently identifies as male and was assigned male at birth (ie, cisgender); (2) be aged ≥18 years; (3) has access to a web-connected device (eg, smartphone, laptop, tablet, or computer); (4) lives in and plans to live in Lima, Peru, for the next 6 months; (5) reports being in a relationship with another cisgender, SMM partner and defines their partner as *someone they feel emotionally or romantically committed to above all others, such as a partner, husband, boyfriend, etc*.; (6) reports being in their relationship for a minimum of 3 months; (7) reports having had CAS with their relationship partner within the previous 3 months; (8) one of the relationship partners reports having had CAS with a casual sex partner in the same timeframe; (9) reports to having no history (ie, within prior 3 months and ever) of intimate partner violence; (10) reports not being coerced by their partner (or others) to participate in the study and related activities; (11) reports feeling safe in the current relationship to participate in the study and related activities; and (12) indicates willingness to test for HIV and other STIs at a local community–based organization at baseline and at 6 months after baseline.

Except for level 1 eligibility criterion number 8, individuals who do not meet all other above-mentioned criteria, will be deemed ineligible, thanked for their interest in the study, and provided a list of HIV and STI prevention resources and other health and relationship-oriented resources that are available in Lima, Peru. Individuals who meet all other criteria (other than number 8) will be prompted to complete an e-consent and enter complete contact information of themselves and their partner. Then, partner 2 will receive a study invite via WhatsApp, which will provide them with access to the eligibility screener and e-consent (if applicable). Level 2 eligibility criteria will automatically be evaluated once both partners complete the eligibility screener.

#### Level 2: Couple-Level Eligibility for Relationship Verification

Web-based relationship verification and validation techniques, which entails a series of questions that compares both the partners’ responses about their relationship (eg, partners’ birth month, tattoo, cohabitation, and pet ownership) will be used [25,26], Relationship verification is based on both partners’ responses to 7 screener items that are automatically evaluated against predetermined decision rules. The 7 items that will be used include age, birth month, relationship status, relationship length, cohabitation, owning pets, and having tattoos. Couples with both partners who provide answers that crossmatch to at least 5 of the 7 questions will be deemed as passing the relationship verification criteria. Couples who do not pass the relationship verification process will not be allowed to participate in the study even if they meet the eligibility criteria of level 1. The relationship verification process of level 2 is in place to help ensure that web-based data will be collected from participants who are in a relationship with one another. Couples who do not pass level 2 eligibility will be thanked for their interest in the study and provided a list of HIV and STI prevention care resources and other health and relationship-oriented resources available in Lima. Couples who meet level 2 eligibility criteria will be invited to attend a brief onboarding Zoom meeting with a staff member of the study.

#### Level 3: Study Onboarding Zoom Meeting

Couples who meet all eligible criteria, provide e-consent, enter complete and accurate contact information, and pass the requirements for *relationship verification and data validation* will be invited to partake in a 10 to 15-minute onboarding Zoom meeting. During the meeting, the study staff will assess the couples’ verification and interest in participating, discuss study expectations and timelines, answer questions, and decide whether to admit the couple into the study. Once admitted, each partner of the couple will be asked to create their own profile on the P3 DHI using their own web-connected device. Their profile will give them access to the baseline assessment followed by whichever intervention group they are randomly assigned to for the pilot RCT.

### Web-Based Fraud Deterrent and Data Validity Safeguards

A variety of fraud deterrents will be embedded and programmed into the eligibility screener to help deter the infiltration of “bots” and fraudulent data entries. Specifically, the eligibility screener will only allow entries from Peru-based IP addresses; no more than 3 screener entries may originate from the same IP address. Start and stop times of completed screener entries will also be monitored and evaluated against a predetermined minimum time for completion. Once an electronically screened individual is deemed eligible for level 1 criteria and has provided e-consent, they will be required to validate their WhatsApp number by entering a 4-digit code sent to their WhatsApp number and entering that specific code in the DHI web system.

Data validity safeguards will also be used to determine whether responses come from 2 unique individuals in a relationship and not from 1 person pretending to be 2 people (ie, fraudulent responses). The eligibility screener date and completion times of couples who pass levels 1 and 2 of the eligibility criteria will also be examined in conjunction with corresponding IP addresses for these entries. Back-to-back screener entries (ie, the start time of the new entry closely following the stop time of the previous entry) and entries specifically from the same IP address will be flagged for review by examining other eligibility criteria and contact information. Overall, each couple must pass the first 2 validation criteria and have no more than 3 screener entries between them. The investigative team uses these relationship verification and data validation test processes to identify true couples in previous and ongoing web-based studies involving couples.

### Ethical Considerations

The FIU and Universidad Peruana Cayetano Heredia Institutional Review Board approved all study-related activities for the 6-month pilot RCT of the P3 DHI (IRB-22-0496, IRB 201101). The project will have a certificate of confidentiality in place as a requirement of the funding agency. The e-consent form will inform participants of confidentiality guidelines and standards. No identifying data will be collected before informed consent is received from the participants; each partner will be required to individually provide consent electronically. The participants will be informed to complete the eligibility screener in a location they deem to be private. If a participant decides to withdraw from the study after providing consent and identifying data (eg, name, email address, and WhatsApp number), then any identifying data associated with the participant will be deleted. In addition, the participants will be instructed to participate in the individual, qualitative exit interview via Zoom or in person at Epicentro in a private setting or a room; the study staff will also be hosting these interviews in a private setting, either on a computer or using a digital recorder. Finally, all correspondence with the participants will be devoid of any indication of the content, purpose, or target population of the study; the participants will be encouraged to delete correspondence (eg, WhatsApp messages) as soon as the participant decides they no longer need the contained information.

Regarding confidentiality and data management practices, neither partner will have access to the other partner’s test results or data from assessments or interviews. Each study participant will be required to create and use a username and unique passcode to gain access and use the P3 DHI. Furthermore, the participants will be automatically logged out of the P3 DHI after a period of inactivity. All data will be coded by a unique individual study number, and personal identifier records will be kept in password-protected files on password-protected computers. HIV and STI test results will be coded using the participant ID; this ID will not have any potential identifying information and will only be accessible to study staff. All recorded videos on Zoom will only occur on the study staff’s secure laptop (password protected and in password-protected files). Similarly, all recorded audio files will only be available on the study staff’s designated digital recorder. From this recorder, the audio will then be immediately uploaded to the study staff’s secure laptop (password protected files). Records will be kept confidential to the level allowed by law, and only the staff assigned to the study will have access to nonanonymous records. Information provided by the study participants will not be released to outside sources unless legally required or written consent is provided by the study participant.

All eligibility screener, consent, and intervention use (ie, paradata) data will be collected and blockchain encrypted via the P3 DHI secure web app hosted on a secure server (Azure; Microsoft Corp). Quantitative baseline and follow-up assessment data will be collected and managed using Qualtrics, an electronic survey data-capturing tool at FIU. Qualtrics is a secure, web-based survey application that is compliant with the Health Insurance Portability and Accountability Act. All Qualtrics servers are backed up daily and monitored for hardware failures. At the end of the trial, all data for the study will be stored on OneDrive, which will only be accessible to the study team through an SSL-encrypted connection that backs FIU’s firewall system.

### Randomization

An allocated randomization block of 2:2, via an embedded algorithm within the P3 DHI, will be used to randomly assign couples to one of the following 2 intervention conditions following the baseline assessment: (1) an immediate intervention condition where couples will be able to use the P3 DHI for 6 months or (2) a 3-month delayed intervention condition where couples will be able to use the P3 DHI for the last 3 months of the trial. For ethical reasons, a delayed control condition was chosen for the trial study to provide all couples with access to the intervention while also permitting the ability to compare outcome differences between trial arms for the first 3 months of the pilot RCT. In sum, all couples in the pilot RCT will have access to use the P3 DHI for a minimum of 3 months, which will enable data pooling of the entire couple sample (N=60; ie, first 3 months for the immediate intervention condition plus the last 3 months with the delayed intervention condition). The study design will also enable the examination of long-term effects of the P3 DHI (ie, effects after 6 months of use) on outcomes and their use of the P3 DHI among couples randomized to the immediate intervention condition (n=30). Randomization will be blinded to couples but not to the study staff (administrators).

### Pilot RCT Baseline and Follow-Up Measures

Items about evaluation will only be assessed during follow-ups after the participants receive the intervention. All other measures, items, and scales will be assessed at each time point throughout the trial, including baseline. In addition to screening for inclusion, the eligibility screener will capture a variety of demographic information from each participant including age, race, sex at birth, gender identity, sexual identity, highest education attainment, current employment status, has a primary health care provider, relationship type, relationship length, couple cohabitation, and couple HIV serostatus. Other demographic information will be captured at each data collection time point (ie, at baseline, months 3 and 6) to assess participant’s current living arrangement, type of health insurance, affordability to pay for health care or medication costs when needed in the past 3 months, as well as whether participants were in a situation that required them to reduce a meal size or skip a meal because of not having enough money for food in the past 3 months (social determinants of health). Skip patterns will be used accordingly for baseline and follow-up assessments at months 3 and 6. Multimedia Appendix 1 [24,27-49] provides a brief description of all other items and scales that will be assessed at each time point and how they relate to the constructs of CIT-HBC [13,14].

Beyond the General Anxiety Disorder 2-item, 5 other items will be used to capture aspects about anxiety and worry specifically related to sex and being in a relationship. The items include the following: “Do you ever feel like you are walking on eggshells around your partner? (Yes, No, I don’t remember)”; “In thinking about the past 3 months, how much did you worry or feel anxious about having sex with your partner? (0=never to 4=always)”; “To what extent did this anxiety stop you from enjoying sex with your partner? (0=not at all to 2=a lot)”; “In thinking about the past 3 months, how much did you worry or feel anxious about having sex with others, not including your partner? (0=never to 4=always)”; and “To what extent did this anxiety stop you from enjoying sex with others, not including your partner? (0=not at all to 2=a lot).” Skip patterns will be used for the above set of items such that some items may not be displayed depending on the participant’s response to the previous item.

Furthermore, some scales will be modified based on feedback from the Community Advisory Board and to help reduce time burden for participants. For instance, single global items from validated scales (eg, Dyadic Trust Scale [34] and Investment Model short form [36]) may alternatively be used to capture interpersonal trust, relationship commitment, and relationship satisfaction instead of the complete scale.

In addition to self-reports of partners using strategies over time, we will ask each participant to test for HIV and STIs at baseline and at 6 months post baseline by going to a local community-based organization. Biological data for new diagnoses of HIV, chlamydia, gonorrhea, and syphilis will be collected. For those previously diagnosed with syphilis, the rapid plasma reagin test will be processed to examine antibodies to syphilis. HIV will be assessed using a rapid test, with a rapid test from another manufacturer used as a confirmatory test if the first is positive. GeneXpert polymerase chain reaction testing will be used to diagnose chlamydia and gonorrhea infections.

### Relationship Dissolution

It is possible that some couples may end their relationship during the eligibility and enrollment process (before enrollment) or sometimes after trial enrollment (eg, between 3 and 6 months after baseline). Couples who self-report that their relationship had ended (through a follow-up assessment or by contacting the study team directly) while participating in the 6-month pilot RCT will not be permitted to continue participating in the study as a couple or as a participant. Couples who end their relationship between assessments will be asked to complete one more assessment, capturing information on any influence they perceive the study to have had on the end of their relationship before being administratively removed from the trial. In an assessment, couples who self-report that their relationship had ended will be administratively removed from the trial afterward. To prevent contamination of the intervention, neither partner of couples who have broken up will be permitted to rejoin the study if or when they enter a new relationship, even if all eligibility criteria are met. We will check this by cross-checking the contact details of all partners in our contact database to ensure that prior participants are not reenrolled.

### Incentives

Each participant will receive an incentive (ie, cash) for their time associated with the study activities at set time points during the pilot trial: baseline 50 Peruvian Soles (US $14) for baseline and 55 Peruvian soles (US $15) for month 3 and month 6, respectively.

### Analytic Plan

#### Overview

For aim 1, we will define and measure feasibility as the ability to recruit, screen for eligibility, enroll, and retain couples in the 6-month pilot RCT over time. Our preliminary work [9-11] demonstrated our ability to recruit, screen for eligibility, and enroll eligible adult cisgender SMM couples in Lima, Peru. We will extend this work for the proposed project to assess feasibility with a larger sample of couples with a focus on retention for the 6-month pilot RCT. As such, some examples of the research questions we seek to answer through this specific aim are as follows: (1) Is it feasible to retain >90% of the couples in the 6-month pilot RCT? (2) Is it feasible to retain >90% of the couples who have both partners complete all 3 quantitative assessments (baseline, month 3, and month 6)? and (3) Is it feasible to have >90% of couples with both partners completing their individual-level, exit interview at 6 months? In line with common research practices (which can help inform a larger RCT), we will evaluate which recruitment strategies (eg, targeted advertisements placed on Facebook or Instagram vs in-person recruitment by research staff) led to the highest proportion of interested individuals starting or completing the eligibility screener as well as couple eligibility and consent. Through our established protocols (ie, standard operating procedures), the study will track and quantitatively describe the reasons why individuals and couples were ineligible, track enrollment rates among consented and eligible couples, assess retention among enrolled couples during the pilot RCT by arm, and which retention practices appealed to participants. The research will also track the reasons for attrition. A detailed CONSORT (Consolidated Standards of Reporting Trials) diagram will be produced at the conclusion of the pilot RCT. We will also quantitatively calculate the proportions of how many participants provided permission and documentation (paper or through the web portal) about their use of prevention strategies during the pilot RCT. We will apply the data obtained from the eligibility screeners to use descriptive statistics at the individual and couple levels and explore whether differences exist based on the couples’ HIV serostatus.

For aim 2, acceptability of P3 will be defined through participant and couples’ use of it (ie, paradata) as well as their attitudes from their experiences of using it over time (ie, quantitative and qualitative dyadic data). For each couple, the research will measure acceptability using and analyzing 3 types of data from both of the relationship partners: (1) quantitative measures will include the Health Information Technology Usability Evaluation scale [24], perceived effect of using P3 on a couples’ relationship [49], and self-reports of the number of intervention modules completed; (2) a qualitative, individual exit interview will be conducted to provide context and supplement partners’ responses to their quantitative measures as well as their top 3 likes, top 3 dislikes, and recommendations to enhance P3; and (3) paradata from the couples’ use of the intervention over time (eg, module completion and time to complete each module).

#### Quantitative Measures

All statistical analyses will be performed with SAS (SAS Institute) or Stata (version 14.2; StataCorp) with modeling using PROC MIXED or GLIMIMIX. We will generate descriptive statistics to calculate means, SDs, counts, and proportions at the individual and couple levels for the Health Information Technology Usability Evaluation scale [24] (mean and SD) and other evaluation items and by dyad HIV serostatus group. The study will also explore, univariately, whether differences exist by couples’ HIV serostatus for these quantitative measures by applying ANOVA and chi-square tests for couple-level continuous and categorical independent variables, respectively.

#### Qualitative Measures

The study will conduct content analysis [50,51] of the qualitative dyadic data collected through the exit interviews at the final follow-up in the pilot trial. Specifically, we will conduct summative content analysis [50,51] of P3 to generate counts and proportions of how many couples had one or both partners mention keywords, topics, or activities, by trial arm, and by couple HIV status. We will also conduct conventional content analysis [50,51] of the qualitative dyadic data to generate coding categories from the transcripts, which may illuminate broader themes of what participants or couples liked and used in P3. Emergent themes may include the use of infographics in educational content about different topics covered, answering questions in the activities to receive tailored recommendations, or watching videos. At least 3 research team members will read all transcripts, take notes, and identify overarching themes. The team will then meet to discuss the overarching themes before rereading and coding the transcripts for these themes, starting with summative content analysis [50,51] to generate counts of keywords before repeating the process for conventional content analysis [50,51]. During subsequent meetings, the team will compare and discuss their coding and will make adjustments as needed before creating the codebook. The codebook will describe the themes and their corresponding definitions. Each team member will then use the codebook to code the transcripts once again. This process will be applied for all transcripts; then, each team member will review one another’s coding of the transcripts to ensure that consistency will be achieved for the themes identified (ie, interrater reliability >0.90). All coding will be done using a web-based management tool, such as Dedoose (9.2.012 Mac; Sociocultural Research Consultants LLC). In the end, a sum count of each individual-level response per theme will be calculated to describe the proportion of endorsement within and between couples for the entire sample, by arm, and by couple HIV serostatus resulting in tables to visualize these endorsements. Representative quotes will also be identified for use to disseminate findings in peer-reviewed outlets (eg, manuscripts and conferences).

#### Paradata From Use of the P3 Intervention

This research will generate descriptive statistics to calculate means, SDs, counts (with ranges), and proportions at the individual and couple levels from the dyadic paradata collected. Firstly, this study will calculate the number of couples who completed all 5 modules in the intervention (vs 4, 3, 2, 1, and none). The researchers will also calculate how much time the participants and couples took to complete each module (eg, module 1=10 minutes) and the total time taken to complete all 5 modules. Next, we will descriptively analyze couples’ responses to the various questions and activities embedded within each of the modules. For all the abovementioned analyses, we will explore whether differences exist by trial arm (eg, n=30 who immediately received the intervention vs n=30 in the delayed control group) and by couples’ HIV serostatus using comparative analyses.

#### Mixed Methods

This research will use an embedded mixed methods design [23] because both quantitative and qualitative dyadic data about the acceptability of the intervention will be collected. In this case, a qualitative strand has been added within a primarily quantitative-focused design (trial assessments and paradata) to enhance our understanding of what participants and couples liked and disliked about the intervention and to provide context about why they appreciated certain features, and, if applicable, what things they wished the intervention had but did not. Put simply, this research will review the findings obtained from analysis of the quantitative data and paradata along with the findings from the qualitative analyses to provide a more comprehensive picture about participants’ and couples’ use and thoughts of P3 relative to their relationships and managing their vulnerability to HIV and other STIs.

#### Aim 3: Preliminary Efficacy of the Intervention

Using the 6-month pilot RCT, this research will examine the preliminary efficacy of the intervention on couples: (A) formation and adherence to an HIV and STI prevention care plan containing evidence-based strategies (eg, ART for U=U, PrEP, and routine testing); (B) formation and adherence to a detailed sexual agreement, (C) relationship functioning (eg, mutual constructive communication and self-efficacy of decision-making and working together), and (D) self-reported and biomarker-confirmed indicators of vulnerability vis-à-vis sexual behaviors (eg, CAS) and HIV and STIs. The pilot RCT will have an allocation ratio of 1:1 and block randomization with a block size of 2, with blocks defined by dyad serostatus. Outcomes A and B will be dichotomous, outcome C will be continuous based on a bounded Likert scales, whereas outcome D will be a count variable based on STI incidence, HIV incidence, and CAS.

Preliminary efficacy will be assessed by considering changes in couples’ outcomes A to D over time. For the immediate intervention arm, the differences in outcomes A to D will be observed between receipt of the intervention and subsequent follow-up assessments at 3 months and 6 months (n=30). For the waitlist arm, the differences in outcomes A to D will be observed between receipt of the intervention after the 3-month assessment and subsequent follow-up assessment at month 6 (n=30). As such, this study will be able to pool and combine data from the 30 couples from the immediate intervention arm with data from the 30 couples from the waitlist arm to observe changes in outcomes (ie, from A-D) over a 3-month time period (N=60). Furthermore, through this study, we will also be able to observe changes in couples’ outcomes (ie, from A-D) for a longer period of time (6 months) for the immediate intervention arm (n=30). Comparisons in couples’ outcomes A to D by trial arm (immediate arm vs waitlist arm) will occur between baseline and month 3. In summary, the design we chose for the proposed 6-month pilot RCT is advantageous for 3 reasons: (1) it will allow us to compare differences between immediate intervention and delayed intervention arms for 3 months; (2) it will allow us to make comparisons within the delayed arm from no intervention before to intervention after (months 3 to 6) the assessment; and (3) it will facilitate generating additional intervention data that can be pooled from the immediate and delayed intervention arms to obtain more robust estimates of intervention effect size to more accurately estimate sample size and power for an efficacy RCT in the future.

#### Primary Analyses

For outcomes A to D, descriptive statistics will be used to characterize the sample at each time point during the trial (baseline, month 3, and month 6), and by trial arm and couples’ HIV serostatus. Descriptive statistics will also be used to summarize cohort characteristics, relationship functioning variables, and sexual behaviors by the trial arm and by couples’ HIV serostatus. For couples, dyadic data will be calculated if there are no missing values from either partner. For continuous variables, couple-level mean variables will be generated by taking the averaged value from both partners’ scores. For within-dyad variables, couple-level differences will be generated by taking the absolute difference between each of the partner’s scores. Categorical dyadic variables will be generated based on whether both partners had the same or different answers. Two-sample 2-tailed *t* tests and chi-square tests will be used to evaluate differences between the immediate intervention arm and delayed intervention control arm for couple-level continuous and categorical independent variables, respectively, to further characterize the sample of couples in the trial.

An intention-to-treat (full analysis) approach will be used to determine the comparison between groups based on the initial randomization and using outcomes for all randomized individuals. The primary analysis will be to compare the proportions of couples with outcomes A, B, and D in the immediate intervention versus the proportions of couples with similar outcomes in the delayed control arm using a chi-square test with one degree of freedom in a univariate analysis from the corresponding contingency table. The efficacy rate will also be presented as the ratio between binomial proportions with CIs estimated through conventional methods used for the couple-level measurement.

Longitudinal analyses will also be conducted [52-54], specifically for outcome C, relationship functioning variables, to explore changes over time and whether these changes differ by arm and by couples’ HIV serostatus. Specifically, generalized linear mixed-effects models (GLMMs) with SAS PROC GLIMMIX will be used to analyze the longitudinal data for outcome C from all 3 time points: baseline, 3 months, and 6 months after randomization. The analysis will fit a GLMM with fixed effects for time, a term for the pilot arm (immediate intervention vs delayed), and an arm-by-time interaction term. The GLMM will include random effects for the intercept and couple cluster (to account for within-couple correlation over time). Model fitting statistics will be used to determine the suitability of more parsimonious (eg, autoregressive) correlation structures and nonlinear effects for time. From the fitted GLMMs, the primary hypothesis will be examined by testing whether the coefficient of the group-by-time interaction term (the difference in slopes between immediate intervention vs delayed) is different from 0. The primary analysis will be performed at the couple level, with exploratory analyses performed at the individual level. For individual-level analyses, all models will use similar GLMMs with random effects to account for the intracluster correlation of couple members. We will also explore outcomes (A-D) at the individual level (n=120) by looking at each participant’s changes over time as secondary analyses of interest using multilevel models.

#### Intention to Treat

An intention to treat approach [55-57] will be used to include all enrolled participants in the analysis. Any individuals or couples lost to follow-up will be assigned an adverse outcome (for outcomes A, B, and D) to be able to include them in the analysis. For outcome C, those lost to follow-up will have to be excluded as the outcome is continuous and there is no cutoff to determine an adverse outcome. This follows an intention-to-treat analysis plan where all randomized individuals are included in the final analysis independent of their participation after baseline assessment, which will also help ensure that the benefit of randomization to balance confounders is retained.

#### Relationship Dissolution

The approximate date and primary reasons for the relationship ending, including whether the reasoning is linked to study participation or intervention use, will be captured through the quantitative follow-up assessment or when the research staff contact participants, each month, to ensure that their contact information is current. To avoid intervention contamination and outcomes, couples who break up (ie, whose relationship has ended) during the pilot RCT will not be permitted to rejoin the study with a new partner. After completion of the pilot RCT, we will conduct a sensitivity analysis to quantitatively explore whether couples who break up comparatively differ from those who did not break up during the pilot RCT. Partners or couples’ data will still be used in the intention-to-treat analysis plan via the last observation carried forward method [55,57]. On the basis of the principal investigator’s prior intervention work with couples, we do not anticipate >5% of couples breaking up during the pilot trial. In the study by Mitchell [8] and another study involving couples [58], <5% of couples had ended their relationship while in the study, and none had reported their reasons as being associated with the study participation. In addition, the length of time that couples will wait before receiving the intervention is 3 months, which may further decrease the possibility of their relationship ending before receiving the intervention. For the reasons mentioned, we expect that the impact of couple dissolution will be minimal in creating systematically different samples between trial arms. However, we cannot predict the rate of relationship dissolution and will adjust analyses accordingly to account for any differences between trial arms and differences over time if observed.

#### Power, Sample Size, and Effect Size Estimations

All power calculations were performed with PASS 2020 [59]. For the couple-level analyses, with 60 couples randomized to 2 arms (30 couples in each), for outcome C, we will have 80% power to detect a difference of 0.75 SDs of the mean relationship functioning measure (large effect [60]) using a linear model. For the dichotomous outcomes A and B, we will have 80% power to detect an absolute difference of 35% between the intervention and delayed conditions using a chi-square test. Our power increases for the outcomes analyzed at the individual level, where the correlation between individuals within a dyad must be considered (ie, the interdependence of individuals nested within dyads). For the incidence rate of outcome D assuming Poisson distribution, 30 couples in each arm will have 80% power to detect a difference of 50% rate reduction in STI rate (including HIV) between the intervention and delayed conditions at 6 months assuming a within-pair coefficient of variation between clusters at 0.25 and the intraclass correlation coefficient as high as 0.1 (even higher ICCs do not reduce rate reduction measure in a significant or meaningful way magnitude wise). For all analyses, the α level was set at .05 with 95% CI. This is a feasibility and acceptability trial that is only powered to detect large effects; however, if effect sizes are lower than hypothesized, we will use that information to properly power a large-scale RCT.

#### Missing Data

Reminders and incentives will be used to maximize participation in the follow-ups. For the primary outcome measures, any missing data due to attrition would be handled as mentioned in the Intention to Treat section. In addition, missing data mechanisms [61-63] will be examined by comparing differences between the dropouts and the observed data. We will perform 25 conditional model-based multiple imputations within group using SAS PROC MI, and will aggregate the results into an overall group, time, and group-by-time interaction with SAS PROC MIANALYZE to provide unbiased intervention effects and their variances. This will also be advantageous if there is a differential dropout for the delayed condition due to wait time. To ensure the validity of the imputed data, 4 parallel models will be performed as a sensitivity analysis on (1) imputed data, (2) completers-only data, (3) assuming adverse outcome for those missing, and (4) applying GLMMs that have the advantage of handling incomplete unbalanced repeated-measures data by incorporating population average and subject-level trajectories, and in turn providing unbiased estimates of parameter of interests. If necessary, post hoc power analyses will be performed using the bootstrap method to calculate the observed power for the detected effect size.

#### Exploratory Secondary Analysis: APIM

To analyze for actor-partner effects using the APIM framework, we will arrange the data in a pairwise format following recommendations provided by Kenny et al [17]. Actor and partner effects will then be estimated using the generalized estimated equations algorithm described by Loeys et al [64], which accounts for the correlation between individuals within a couple using a robust variance estimate. The Wald test based on robust variance of the generalized estimated equations models performs quite well in testing actor and partner effects when the number of dyads is >50 [65]. Several models will be examined for significant actor-partner effects about cisgender SMM couples’ outcomes of A and B. For each outcome, 2 actor-partner models will be constructed to test the impact of demographics and common relationship characteristics (model 1) separately from the impact of the average absolute change in relationship functioning variables (eg, couple mean and within-dyad difference) over time (model 2). Trial arm assignment will be controlled in each actor-partner model as a potential confounder. The parameter estimates produced from these models will be used to detect the actor-partner effects. To make the interpretation of the actor-partner effects more intuitive, we will convert the coefficients and 95% confidence limits to adjusted relative risk ratio and corresponding 95% confidence limits for presentation. Two-sided *P* values <.05 will be considered statistically significant. We will also consider analyzing joint and mutual joint effects based on time availability.

## Results

This 6-month pilot RCT is ongoing. Recruitment, enrollment, and data collection began in January 2023 and ended in April 2024. A total of 74 adult cisgender SMM couples met all inclusion criteria, provided consent, and were enrolled in the pilot RCT. Retention was 92% (68/74) at month 6. Data are currently being analyzed to address the 3 specific aims. Findings will be disseminated through peer-reviewed journals and conference presentations.

## Discussion

### Principal Findings

This protocol describes the methods that will be used to conduct a 6-month pilot RCT to assess the feasibility, acceptability, and preliminary efficacy of P3, a couples-based DHI. Regarding feasibility, the enrolled sample exceeded the targeted sample size of 60 adult cisgender SMM couples by 23% (74/60), indicating a clear interest in this type of intervention and potential future public health service for the local population in Lima, Peru. Future findings from the trial will reveal details pertaining to which recruitment strategies worked best to reach the intended audience, how well the enrollment procedures worked, and the proportion of couples who were retained at the end of the 6-month pilot RCT along with reasons for attrition.

Findings relevant to the acceptability of P3 will be showcased using various data sources, all indicating whether couples deemed P3 to be acceptable as a couples-based DHI for meeting their prevention-care and relationship needs. Acceptability-related findings will also help describe potential areas to refine and improve P3.

It is anticipated that results from the pilot RCT will reveal the preliminary efficacy of the P3 DHI on couples’ formation and adherence to an HIV and STI prevention-care plan containing evidence-based strategies (eg, routine testing, PrEP, and ART for U=U), formation and adherence to a detailed sexual agreement, and their perceptions that their prevention-care plan and sexual agreement were in alignment. In addition, preliminary efficacy results will highlight whether couples’ relationship functioning, including mutual constructive communication and self-efficacy in working and making decisions together about their sexual health, improved over time. Finally, preliminary efficacy results will describe changes regarding partners’ and couples’ vulnerability to HIV and other STIs over time by analyzing self-reports of sexual behaviors (eg, CAS) and biomarker confirmation of test results.

### Conclusions

The protocol details the methods of how the 6-month pilot RCT will be conducted and the expected findings from it. The ultimate goal of this line of research is to complement and expand HIV and STI prevention-care services by offering an effective couples-based DHI for adult cisgender SMM couples in Lima, Peru. Couples-based HIV serostatus neutral programs currently do not exist, despite the public health need for such research [6]. The anticipated next step of this research will include conducting a fully powered RCT to establish the efficacy of the P3 DHI. In addition, future research is needed to better understand the real-world barriers and facilitators of implementation and dissemination to sustainably offer P3 DHI within the realm of existing HIV- and STI- related services. As such, the next study would include conducting a hybrid type 1 trial [66,67] along with integrating the findings and lessons learned from this pilot trial.

## Appendix

Multimedia Appendix 1 Brief description of scale or items relative to couples interdependence theory for health behavior change construct.

Multimedia Appendix 2 Peer review report from HIV/AIDS Intra- and Inter-personal Determinants and Behavioral Interventions Study Section - Risk, Prevention and Health Behavior Integrated Review Group - Center for Scientific Review (National Institutes of Health, USA).

## Abbreviations

ADAPT-ITT: Assessment, Decision, Adaptation, Production, Topical Experts, Integration, Training, and Testing
APIM: actor-partner interdependence model
ART: antiretroviral therapy
CAS: condomless anal sex
CIT-HBC: Couples Interdependence Theory for health behavior change
CONSORT: Consolidated Standards of Reporting Trials
DHI: digital HIV serostatus-neutral intervention
FIU: Florida International University
GLMM: generalized linear mixed-effects model
P3: Para Ti, Para Mí, Para Nosotros
PrEP: pre-exposure prophylaxis
RCT: randomized controlled trial
SMM: sexual minority men
STI: sexually transmitted infection
U=U: undetectable=untransmissible
UPCH: Universidad Peruana Cayetano Heredia

## Data Availability

The data sets generated during and analyzed during this study are available from the corresponding author on reasonable request.
