# Supplementary material for: Assessing a Couples-Based, Digital HIV Serostatus-Neutral Intervention (Para Ti, Para Mí, Para Nosotros) for Adult Cisgender Sexual Minority Male Couples in Lima, Peru: Protocol for a 6-Month Pilot Randomized Controlled Trial
Source: JMIR Res Protoc. 2024 Oct 10;13:e63106. doi: 10.2196/63106 (PMC11502978; doi:10.2196/63106)
Supplement: Multimedia Appendix 1 [file resprot_v13i1e63106_app1.docx]

Table S1. Brief description of scale or items relative to couples interdependence theory for health behavior change (CIT-HBC) construct.

| CIT-HBC construct and scale or item | | Brief description | Number of items | Response range | Cronbach α |
| --- | --- | --- | --- | --- | --- |
| **Predisposing factors of the couple** | | | | | |
|  | CLMS^a^ scale: Couple-level stigma subscale [26] | Perceptions of anticipated stigma in various contexts and situations because of being a same-sex couple | 6 | 0=not at all to 4=a great deal | 0.89 |
|  | CLMS scale: couple-level discrimination subscale [26]^b^ | Recollection of discrimination experiences in various contexts and situations because of being a same-sex couple | 5 | 0=never to 4=always | 0.94 |
|  | CLMS scale: couple-level visibility subscale [26] | Recollection of avoidance, misrepresentation, and/or challenges in different situations because of being a same-sex couple | 5 | 0=never to 4=always | 0.89 |
|  | CLMS scale: Lack of social support for couples subscale [26] | Perceptions of social support available to them as a same-sex couple | 4 | 0=not at all true to 4=completely true | 0.75 |
|  | CLMS scale: Lack of integration with families of origin subscale [26] | Recollection of acknowledgment, invitations, and general inclusion of one’s partner in family-related events | 6 | 0=not at all true to 4=completely true | 0.91 |
|  | General disclosure of sexual identity scale [27] | Captures how open one is about their sexual orientation to family, friends, coworkers, and health care providers | 4 | 1=not open at all to 5=open to almost everyone | 0.82 |
|  | Internalized homophobia [28] | Assessed perceptions about internalized stigma regarding one’s sexual identity of being gay or bisexual | 2 | 1=strongly disagree to 5=strong agree | N/A^p^ |
|  | LGBT^c^ Connectedness scale [29] | Measures perceptions about their connectedness with the LGBT community | 8 | 1=strongly disagree to 4=strongly agree | 0.81 |
|  | Sexual agreements [30] | Type, explicitly discussed, permitted behaviors and adherence | 11 | Response options vary by item | N/A^p^ |
|  | Sexual agreement investment scale [31]^b^ | Global items to assess overall value, commitment, and satisfaction of one’s current sexual agreement with their partner | 3 | 0=not at all, 1=a little, 2=moderately, 3=very much, and 4=extremely | N/A^p^ |
|  | Importance of sexual agreement communication scale [32] | Assesses communication relative to current sexual agreement with partner | 5 | 0=not at all to 4=extremely | 0.84 |
|  | Sexual agreement self-efficacy scale [32]^b^ | Assesses self-efficacy of honoring current sexual agreement with partner | 1 | 0=not at all to 4=extremely | 0.92 |
|  | Relationship dynamics of interpersonal trust, relationship commitment, and relationship satisfaction [33-35]^b^ | Global items were used to capture perceived trust of partner, commitment to current relationship, and general satisfaction with current relationship. | 3 | 0=not at all to 4=completely | N/A^p^ |
|  | Mutual constructive communication patterns subscale [36-38] | Participants’ perceptions of how they and their partner typically deal with problems in the relationship | 3 | 1=very unlikely to 9=very likely | 0.71-0.75 |
|  | Relationship Values Scale [39]^b^ | Participants’ perceptions about equality and intimacy regarding their partner and the relationship | 5 | 1=not at all true to 9=very true | 0.91 |
|  | PHQ-2^d^ [40,41] | Screens for mental well-being or depression | 2 | 0=not at all to 3=nearly every day | 0.81 |
|  | GAD-2^e^ [41,42] | Screens for generalized anxiety disorder | 2 | 0=not at all to 3=nearly every day | 0.82 |
|  | Godin-Shephard Leisure-Time Physical Activity Questionnaire [43] | Captures the number of times during a typical 7-day period that one exercised for ≥15 minutes (strenuous, moderate, or mild) during the previous 3 months | 3 | Numeric | N/A^p^ |
|  | AUDIT-C^f^ [44] | Screens for hazardous alcohol use disorders | 3 | Response options vary by item | 0.90 |
|  | Substance use | Captured the use of cannabis, cocaine, inhalants (eg, poppers), or other drugs during the past 3-months without a prescription  The final item assessed whether use of any reported substance led to health, social, legal or financial problems. | 5 | 0=never, 1=less than monthly, 2=monthly, 3=weekly, and 4=daily or almost daily | N/A^p^ |
|  | Belief and worry about acquiring HIV | Perceived frequency of worry and likelihood of acquiring HIV in lifetime | 2 | Worry: 0=never to 4=always Likelihood: 1=very unlikely to 4=very likely | N/A^p^ |
|  | Sexual risk behaviors | Number of anal acts by role, CAS^g^, partner type, and partner’s HIV status | 3-9 | Numeric | N/A^p^ |
|  | HIV serostatus [45] | Self-report, individual, and couple | 2 | Categorical | N/A^p^ |
|  | Prior testing history [45] | Frequency of HIV or STI^h^ testing | 2 | Categorical | N/A^p^ |
|  | PrEP^i^ awareness and experience | Items captured general awareness, experience, medication adherence in the past 30 days, the worry about what others think that prevented PrEP disclosure, reasons for stopping PrEP, and worry about what others think that prevented PrEP uptake | 6 | Response options vary by item | N/A^p^ |
|  | HIV care [46] | Months living with HIV; number of physician visits in the past 12 months; next scheduled appointment; ART^j^ status; ART dose adherence in prior 7 days; viral load test in the past 12 months and result; frequency of following HIV medication schedule and special instructions; ART adherence during the previous weekend; and perceived partner support for medication adherence | 11 | Response options vary by item | N/A^p^ |
| **Partner’s transformation and motivation** | | | | | |
|  | Partner’s transformation and motivation [47]^b^ | Ability of the participants to respond (1) cognitively and (2) emotionally to the health threat of HIV or STIs by valuing the we or us in the relationship over the me or I | 1 |  | N/A^p^ |
| **Process and use of communal coping** | | | | | |
|  | Outcome efficacy to reduce HIV or STI vulnerability [47]^b^ | Belief that both the partners’ sexual agreement type and HIV prevention and care strategy use align in their relationship | 2 | — | N/A^p^ |
|  | Couple efficacy to reduce HIV or STI vulnerability [47]^b^ | Use strategies of couple communication, planning and decision-making, and joint effort to having detailed sexual agreement and using HIV prevention and care strategies in the relationship | 3 | — | N/A^p^ |
| **Initiate and maintain health-enhancement behaviors** | | | | | |
|  | Participants’ attitudes about, support for and current use of evidence-based strategies of HIV prevention care | Routine HIV testing (3 or 6 months); routine HIV testing (12 months); Routine STI testing (3 or 6 months); Routine STI testing (12 months); encouraging relationship partner to test; encouraging other sex partners to test; discussing HIV status and STI status with the partner; discussing HIV status and STI status with other sex partners; making an explicit agreement with the partner about whether to be monogamous or permit sex with others; daily oral PrEP; PrEP 2-1-1; injectable PrEP; nPEP^k^; always using condoms with the partner; occasionally using condoms with the partner; always using condoms with other sex partners; occasionally using condoms with other sex partners; perception of not using condoms if both the partners are HIV negative and monogamous; perception of not using condoms if both the partners are HIV negative and on PrEP; perception of not using condoms if my partner is living with HIV, consistently taking ART, and virally suppressed (U=U^l^); perception of not using condoms if I am living with HIV, consistently taking ART, and virally suppressed (U=U); and consistently taking ART to achieve viral suppression | 22 | 0=don’t want to use, 1=currently using, 2=does not apply, and 3=not using but would in the future | N/A^p^ |
|  | Perception of partner’s responses to items regarding attitudes, support for, and current use of evidence-based strategies for HIV prevention care | Routine HIV testing (3 or 6 months); routine HIV testing (12 months); routine STI testing (3 or 6 months); routine STI testing (12 months); encouraging relationship partner to test; encouraging other sex partners to test; discussing HIV status and STI status with partner; discussing HIV status and STI status with other sex partners; making an explicit agreement with the partner about whether to be monogamous or permit sex with others; daily oral PrEP; PrEP 2-1-1; injectable PrEP; nPEP; always using condoms with partner; occasionally using condoms with partner; always using condoms with other sex partners; occasionally using condoms with other sex partners; no condoms for us if partner and I are HIV negative and monogamous; no condoms for us if partner and I are HIV negative and on PrEP; no condoms for us if my partner is living with HIV, consistently taking ART, and virally suppressed (U=U); no condoms for us if I am living with HIV, consistently taking ART, and virally suppressed (U=U); and consistently taking ART to achieve viral suppression | 22 | 0=don’t want to use, 1=currently using, 2=does not apply, and 3=not using but would in the future | N/A^p^ |
| **Evaluation of P3**^m^ **DHI**^n^ | | | | | |
|  | Health-ITUES^o^ [48] | Evaluation scale for digital health: considers impact, perceived usefulness, perceived ease of use, and user control | 20 | 1=strongly disagree to 5=strongly agree | 0.85-0.92 |
|  | Perception of research participation [49]^b^ | Items assess perceived effects from using P3 on their relationship, ranging from discussions to identifying strengths and weaknesses | 5 | 1=not at all or very negative to 9=a lot or very positive | N/A^p^ |
| **Reduce weakness** | | | | | |
|  | Assessing for possible contamination between trial arms | Assess whether participants have discussed the study with others while in the trial or heard from others that they are also in the study | 2 |  | N/A^p^ |

^a^CLMS: Couple-level Minority Stressor.

^b^Scale or items that will be modified for cultural relevance and/or to help reduce participant burden (ie, to reduce survey length).

^c^LGBT: lesbian, gay, bisexual, transgender.

^d^PHQ-2: Patient Health Questionnaire-2.

^e^GAD-2: Generalized Anxiety Disorder-2.

^f^AUDIT-C: Alcohol Use Disorders Identification Test – Consumption.

^g^CAS: condomless anal sex.

^h^STI: sexually transmitted infection.

^i^PrEP: pre-exposure prophylaxis.

^j^ART: antiretroviral therapy.

^k^nPEP: non-occupational post-exposure prophylaxis.

^l^U=U: undetectable=untransmissible.

^m^P3: Para Ti, Para Mí, Para Nosotros.

^n^DHI: digital HIV serostatus-neutral intervention.

^o^Health-ITUES: Health Information Technology Usability Evaluation scale.

^p^N/A: not applicable.
