# Supplementary material for: Assessing a Couples-Based, Digital HIV Serostatus-Neutral Intervention (Para Ti, Para Mí, Para Nosotros) for Adult Cisgender Sexual Minority Male Couples in Lima, Peru: Protocol for a 6-Month Pilot Randomized Controlled Trial
Source: JMIR Res Protoc. 2024 Oct 10;13:e63106. doi: 10.2196/63106 (PMC11502978; doi:10.2196/63106)
Supplement: Multimedia Appendix 2 [file resprot_v13i1e63106_app2.pdf]

SUMMARY STATEMENT
(Privileged Communication)

PROGRAM CONTACT:
Gregory Greenwood
240-669-5532
gregory.greenwood@nih.gov

Release Date: 04/04/2021
Revised Date:

Application Number: 1 R34 MH126776-01A1
Formerly: 1R34MH126776-01

Principal Investigator

MITCHELL, JASON W

Applicant Organization: FLORIDA INTERNATIONAL UNIVERSITY

Review Group: HIBI
HIV/AIDS Intra- and Inter-personal Determinants and Behavioral Interventions Study Section
AIDS - EXP. REV.

Meeting Date: 03/15/2021
Council: MAY 2021
Requested Start: 07/01/2021
RFA/PA: PA20-141
PCC: 9A-ASPT

Project Title: Acceptability, feasibility, and preliminary impact of a web-based, HIV prevention toolkit with cisgender male couples in Lima, Peru
SRG Action: Impact Score:27 Percentile:13 +
Next Steps: Visit https://grants.nih.gov/grants/next_steps.htm
Human Subjects: 30-Human subjects involved - Certified, no SRG concerns
Animal Subjects: 10-No live vertebrate animals involved for competing appl.
Gender: 3A-Only men, scientifically acceptable
Minority: 5A-Only foreign subjects, scientifically acceptable
Age: 3A-No children included, scientifically acceptable

Project Year	Direct Costs Requested	Estimated Total Cost
1	150,000	205,375
2	150,000	205,375
3	150,000	205,375
TOTAL	450,000	616,124

ADMINISTRATIVE BUDGET NOTE: The budget shown is the requested budget and has not been adjusted to reflect any recommendations made by reviewers. If an award is planned, the costs will be calculated by Institute grants management staff based on the recommendations outlined below in the COMMITTEE BUDGET RECOMMENDATIONS section.

1R34MH126776-01A1 Mitchell, Jason

RESUME AND SUMMARY OF DISCUSSION: This application proposes to continue the team's efforts to adapt a couples-based HIV/STI prevention toolkit intervention for use with cisgender male couples in Lima, Peru using the ADAPT-ITT model with elements of human-centered design. The adapted intervention will be tested in pilot RCT with 60 male couples assigned to the intervention or the waitlist control. Outcomes will be retention/attrition rates and the acceptability of the toolkit intervention. The preliminary impact of the toolkit intervention on the couple's use of HIV prevention strategies over time will also be assessed. The committee felt that the proposed adaption of the intervention was innovative in that the new content will focus notable elements of the Peruvian context such as the importance of family relationships. The team is strong and they have presented preliminary data supporting the acceptability of the intervention in Peru. The team has been highly responsive to concerns raised in the prior review of this application. They have published the pilot study findings, addressed concerns about significance and innovation. In addition, the team has provided details about the measures, use of theater testing, adjusted Aim 1, and expanded the discussion of the proposed analyses. The committee noted a few remaining minor methodological questions, but these questions did not diminish the committee's overall high enthusiasm for the many strengths of this responsive resubmission.

DESCRIPTION (provided by applicant): Sexual minority men in Lima, Peru are disproportionately impacted by HIV, and a substantial proportion of these 4 men acquire HIV while in a relationship (i.e., cisgender male couples). Studies conducted by members of the 5 investigative team found that cisgender male couples' use of evidence-based HIV/STI prevention strategies (e.g., 6 ART, PrEP, routine HIV/STI testing, condom use) are sub-optimal to reliably prevent acquisition and/or 7 transmission of HIV and other STIs given their current engagement in sexual risk behaviors. To date, no couples- 8 based HIV/STI prevention interventions are available in Lima or elsewhere in Peru. The overarching goal of the 9 proposed 3-year project is to help fill this critical gap in Peru's HIV/STI prevention services. To start, we 10 conducted a mixed method pilot project to assess the acceptability of the PI's theoretically-grounded eHealth, 11 couples-based HIV/STI prevention toolkit intervention with 42 cisgender male couples in Lima (14 concordant 12 HIV-negative, 16 discordant, 12 concordant HIV-positive). 90% of couples reported high acceptability of the 13 intervention, and liked the agreement builder activity the most. Couples told us they wanted more information 14 about HIV treatment as prevention (PrEP, ART, TasP/U=U) and how to strengthen and manage their own and 15 familial relationships (cultural context). They also wanted the intervention to be accessible on different web- 16 connected devices. Additional findings revealed only 33%-50% of couples included evidence-based HIV/STI 17 prevention strategies in their agreement. To help improve couples' uptake and continued use of evidence-based 18 prevention strategies, our solution is to integrate an electronic algorithm into the agreement builder activity to 19 provide them with tailored recommendations that align with their engagement in sexual behaviors and 20 relationship profile. Methods: Via the proposed R34, our next steps are to apply the remaining stages of the 21 ADAPT-ITT model with elements of human-centered design to adapt our couples-based intervention. We will 22 then conduct a 6-month pilot RCT with a waitlist control to assess the feasibility, acceptability, and preliminary 23 impact of the adapted intervention with 60 cisgender male couples, stratified by their dyad HIV serostatus, on 24 outcomes of: a) formation and adherence to a tailored risk-reduction plan and agreement; b) relationship 25 functioning (e.g., communication); c) use of evidence-based HIV prevention strategies; and d) HIV/STI incidence 26 over time. Our proposed approach is rigorous with scientific premise as mixed methods and a pilot RCT with 27 a waitlist control will be used to achieve our Specific Aims. Our project is also timely and practical with key 28 stakeholder buy-in, suggesting the high Public Health significance of our proposal. Our findings will reveal the 29 feasibility, acceptability and preliminary impact of the adapted toolkit intervention to reduce couples' HIV/STI risk 30 via sustained use of evidence-based HIV prevention strategies over time through their formation and adherence 31 to a

tailored agreement and risk-reduction plan. Our next step will be to apply these findings (e.g., R01) to test 32 the intervention for efficacy. Our project is of interest to NOT-MH-20-019 and NOT-MH-20-020.

PUBLIC HEALTH RELEVANCE: We propose to conduct a novel, 3-year project to adapt an existing, theoretically guided eHealth couples-based HIV/STI prevention toolkit intervention to meet the specific needs of cisgender male couples in Lima, Peru by applying the ADAPT-ITT framework with elements of human-centered design. Following adaptation, which will include cultural aspects, a 6-month pilot RCT with a waitlist control will be conducted to assess feasibility, accessibility and preliminary impact of the toolkit intervention on couples': a) formation and adherence to a tailored risk-reduction plan and agreement; b) relationship functioning (e.g., communication); c) use of evidence- based HIV prevention strategies; and d) HIV/STI incidence over time. The proposed research is rigorous with scientific premise, has high Public Health significance, and will lead to findings to help cisgender male couples reduce their risk for acquisition and/or onward transmission of HIV/STIs through a tailored and accessible prevention intervention.

CRITIQUE 1

Significance: 2
Investigator(s): 1
Innovation: 3
Approach: 3
Environment: 1

Overall Impact: This resubmission proposal addresses a significant issue in the field of HIV prevention among cisgender male couples in Peru. The approach is strong as it follows the ADAPT-ITT model and includes a randomized controlled trial with a waitlist control. An advisory board will be used for the adaptation of the intervention. The project is innovative in that is adapting an evidence-based intervention to a new setting and population. The team has strong preliminary data and prior experience working together and with this population. The revisions were responsive to the original review and hence the application is significantly stronger. There was one minor weakness in aim 3 due the inclusion of several outcomes without establishing the primary outcome. The proposal's impact is potentially high given the significant rates of HIV and STI's among sis-gender MSM in Peru and preliminary evidence of the impact of couple based interventions in increasing adherence to HIV/STI prevention evidence based strategies.

1. Significance:

Strengths

- The proposed study addresses a significant gap in HIV prevention in Peru: The availability of culturally congruent behavioral interventions for sis-gender male couples
- This is a marginalized population that bares significant disparities in HIV infection and access to high quality couples' interventions
- If successful, the intervention is likely to have a significant impact increasing adherence to evidence-based HIV treatment and prevention
- The proposal provides promising findings from a previous pilot study examining acceptability of the intervention in Peru

- The proposal is grounded in rigorous literature that supports the need for the intervention and its promise.
- The intervention is informed by Couples Interdependence Theory

Weaknesses

- None noted by the reviewer

2. Investigator(s):

Strengths

- The investigative team is experienced and has conducted extensive research in HIV prevention in Peru. They developed the intervention to be adapted in the current proposal

Weaknesses

- None noted by the reviewer

3. Innovation:

Strengths

- The proposal aims to adapt an evidence-based couples HIV prevention intervention to a new population and setting
- Leveraging algorithms to provide personalized recommendations to each couple is innovative

Weaknesses

- None noted by the reviewer

4. Approach:

Strengths

- The use of the ADAPT-ITT model with elements of the human centered design strategy to guide the intervention adaptation is a strength
- The approach uses a combination of qualitative and quantitative methods to examine acceptability, feasibility, and potential effects of the intervention
- The adaptation strategy is thorough and appropriate
- Recruitment and retention plans were detailed, appropriate, and well justified
- A community advisory board will inform the intervention adaptation
- Addressing STIs and HIV simultaneously is a strength

Weaknesses

- Aim 3 does not separate primary outcome from secondary ones according to the theoretical framework. It is unclear which outcome will be used for estimates of effects
- Outcomes need to be more specifically operationalized, particularly HIV and STI outcomes

5. Environment:

Strengths

- The partnership between Florida International University, the Universidad Peruana Cayetano Heredia, UCLA, and Espacio Comun provide the necessary resources and expertise for the completion of this project

Weaknesses

- None noted by the reviewer

Study Timeline:

Strengths

- The study's timeline accounts for all activities necessary to conduct the study and allocates sufficient time to complete it

Weaknesses

- None noted

Protections for Human Subjects:

Acceptable Risks and/or Adequate Protections

- Appropriate risk/benefit analysis. This revision enhanced human subjects protections by outlining more detailed procedures handling IPV/ coercion that may be detected in the course of the study

Data and Safety Monitoring Plan (Applicable for Clinical Trials Only):

Acceptable

- Included plans to set up a board and monitor the handling of human subjects in the study

Inclusion Plans:

- Sex/Gender: Distribution justified scientifically
- Race/Ethnicity: Distribution justified scientifically
- For NIH-Defined Phase III trials, Plans for valid design and analysis: Not applicable
- Inclusion/Exclusion Based on Age: Distribution justified scientifically
- Includes people 18 and older
- Includes only cisgender MSM because they are disproportionately impacted and including transgender populations go beyond the scope of the current study
- Includes all racial groups

Vertebrate Animals:

Not Applicable (No Vertebrate Animals)

Biohazards:

Not Applicable (No Biohazards)

Resubmission:

- Responsive to prior review

Applications from Foreign Organizations:
Not Applicable (No Foreign Organizations)

- The study population in Peru carries a disproportionate burden of HIV infections

Select Agents:
Not Applicable (No Select Agents)

Resource Sharing Plans:
Acceptable

- Includes appropriate plans for data sharing and dissemination

Authentication of Key Biological and/or Chemical Resources:
Not Applicable (No Relevant Resources)

Budget and Period of Support:
Recommend as Requested

CRITIQUE 2

Significance: 1
Investigator(s): 1
Innovation: 2
Approach: 2
Environment: 1

Overall Impact: This highly responsive resubmission application proposes to conduct a pilot RCT to evaluate a theory-informed couples-based eHealth HIV/STI prevention toolkit for cisgender MSM in Lima, Peru by assessing intervention feasibility, acceptability and preliminary efficacy for developing/adhering to couples' risk reduction plan, relationship functioning, use/adherence to of HVI/STI prevention strategies, and HIV/STI incidence. Prior to the pilot RCT, the intervention will be adapted based on findings from the investigative team's preliminary research and, following the remaining stages of the ADAPT-ITT framework, engage potential end-users and other experts to finalize adaptations. The study is highly significant in its focus on MSM couples in Lima given that MSM are disproportionately burdened by HIV/STIs and many new HIV infections among MSM are acquired from primary relationship partners. The application is innovative in its incorporation of content that reflects the Peruvian context including the importance of family relationships. It may be the first couples-based intervention for MSM to include a focus on family relationships. If shown to be a critical intervention element, it may have implications for couples-based HIV/STI prevention interventions for MSM elsewhere. Minor weaknesses in the approach include uncertainty about the ability to detect differences in HIV/STI incidence between arms in a 6-month period and ability to recruit highest risk

couples for participation. In spite of these weaknesses, this proposed research has the potential to have a high impact on the field.

1. Significance:

Strengths

- The proposed intervention addresses a high priority population in Peru as cisgender MSM are disproportionately affected by HIV/STIs.
- It is of high significance because the intervention has the potential to help address one of the key ways in which MSM in Peru acquire HIV, from their primary relationship partner (~33%).
- eHealth-delivered, couples-based HIV/STI interventions have the potential to simultaneously improve communication, commitment to reducing risk and address other relationship factors as well as increase uptake of HIV/STI prevention strategies by introducing those most likely to be appealing for each couple.
- The investigative team has piloted the couples-based intervention with MSM in Peru and it was found to be highly acceptable.
- Initial intervention adaptations are guided by rich feedback/suggestions from participants in the prior pilot study. One key intervention adaptation will attend to MSM couples' need for support in balancing their relationships with their primary partner and their family.

Weaknesses

- None noted.

2. Investigator(s):

Strengths

- PI Mitchell has a wealth of expertise designing, implementing, and testing eHealth-delivered couples-based prevention interventions for MSM that address relationship dynamics and HIV/STI prevention strategy uptake/adherence. Mitchell has both quantitative and qualitative analysis skills and experience conducting research with MSM in Peru.
- Co-Is Konda and Silva-Santisteban, both based in Peru, also have expertise in behavioral and biomedical HIV/STI prevention with MSM, conducting combination prevention clinical trials in Peru, and collection of biomedical and behavioral data. Konda also collaborated with Mitchell on the initial pilot of the intervention in Peru and brings additional qualitative analysis expertise.

Weaknesses

- None noted.

3. Innovation:

Strengths

- The proposed study will incorporate content/changes requested by MSM pilot participants reflective of the Peruvian context (e.g., assistance navigating primary partner/family relationships).
- The adapted intervention may be the first couples-based intervention to address family relationships among MSM.

- Using algorithms to tailor HIV/STI prevention strategies based on couples' unique characteristics is innovative and may increase uptake of strategies.

Weaknesses

- None noted.

4. Approach:

Strengths

- The development of a CAB, which includes MSM couples, to assist with the adaptation phase increases the likelihood adaptations will be acceptable end-users.
- The human-centered design methodologies and iterative approach that will be used to adapt the intervention further increase the likelihood of acceptable adaptations.
- A final testing stage will provide an opportunity to test all study procedures and identify any last modifications needed to the intervention prior to the pilot RCT.
- Collection and analysis of quantitative, qualitative, and paradata to assess acceptability is a strength.
- Strategies to ensure scientific rigor clearly defined.
- Collaboration with Epicentro, where baseline and final follow-up appointments will be conducted, is a strength as it will facilitate access to HIV/STI prevention/treatment as needed.

Weaknesses

- Unclear if the proposed recruitment strategies will be effective in reaching/enrolling highest risk MSM couples.
- Although not a primary outcome, it is unclear if 6 months will be long enough to see changes in HIV/STI incidence between study arms.

5. Environment:

Strengths

- The research environment (Florida International University, UPCH, UCLA, Epicentro) for the proposed research is strong and will support the conduct of this study.

Weaknesses

- None noted.

Study Timeline:

Strengths

- Timeline is appropriate for the activities proposed in this research.

Weaknesses

- None noted.

Protections for Human Subjects:

Acceptable Risks and/or Adequate Protections

Data and Safety Monitoring Plan (Applicable for Clinical Trials Only):
Acceptable

Inclusion Plans:

- Sex/Gender: Distribution justified scientifically
- Race/Ethnicity: Distribution justified scientifically
- For NIH-Defined Phase III trials, Plans for valid design and analysis: Not applicable
- Inclusion/Exclusion Based on Age: Distribution justified scientifically

Vertebrate Animals:
Not Applicable (No Vertebrate Animals)

Biohazards:
Not Applicable (No Biohazards)

Resubmission:

- This resubmission application is highly responsive to prior critiques.

Applications from Foreign Organizations:
Not Applicable (No Foreign Organizations)

- Foreign Organization included but is not the applicant organization

Select Agents:
Not Applicable (No Select Agents)

Resource Sharing Plans:
Acceptable

Authentication of Key Biological and/or Chemical Resources:
Not Applicable (No Relevant Resources)

Budget and Period of Support:
Budget Modifications Recommended (in amount/time)
Recommended budget modifications or possible overlap identified:

- Unclear if $25,000 to the eHealth developer will be sufficient for scope of adaptations proposed.

CRITIQUE 3

Significance: 2
Investigator(s): 1
Innovation: 2
Approach: 2
Environment: 2

Overall Impact: This revised application seeks to build off prior work to adapt and pilot test a couples-based intervention in Lima, Peru. The investigators are strong, with the PI having a long record of research in MSM couples. The work is innovative in that HIV transmission is often treated as an individual behavior, though a significant proportion of HIV infections are attributable to main sex partners (dyads).The application was responsive to prior critique.

1. Significance:

Strengths

- For HIV to be transmitted, a minimum of two partners are needed. Yet much HIV research is focused on individuals. Data suggest a significant portion of HIV is transmitted in the context of a committed relationship
- No research on MSM couples in Lima, Peru
- Limited research on MSM couples
- HIV remains a significant issue for MSM, it is 10x higher among MSM than the general population

Weaknesses

- None noted

2. Investigator(s):

Strengths

- PI team is strong. Mitchell has a long record of doing HIV research with MSM couples

Weaknesses

- None noted

3. Innovation:

Strengths

- There are no couples-based MSM HIV interventions in Lima Peru
- The study leverages eHealth and algorithms
- The intervention includes family and familial perspectives (key for the population to be studied)

Weaknesses

- None noted

4. Approach:

Strengths

- Study is built off prior work
- Study is theoretically-grounded in eHealth
- Work has already begun, the team seeks R34 funding to use an established method ADAPT-ITT to refine the intervention and assess feasibility and acceptability.
- The recruitment and retention plan seems robust

Weaknesses

- None noted

5. Environment:

Strengths

- Local partners in Peru enhances chances of success
- FIU has adequate resources to support the PI

Weaknesses

- None noted

Study Timeline:

Strengths

- The study timeline accounts for most activities to be conducted and all are within the allocated 36M timeline

Weaknesses

- Greater detail regarding recruitment for the pilot study versus follow up would have been appreciated. We see when the pilot is being "tested" which presumably means recruitment and follow up, but it would be nice to know when the team expects to start and stop recruitment. This is not a score driving concern.

Protections for Human Subjects:

Acceptable Risks and/or Adequate Protections

Data and Safety Monitoring Plan (Applicable for Clinical Trials Only):

Acceptable

Inclusion Plans:

- Sex/Gender: Distribution justified scientifically
- Race/Ethnicity: Distribution justified scientifically
- For NIH-Defined Phase III trials, Plans for valid design and analysis: Not applicable
- Inclusion/Exclusion Based on Age: Distribution justified scientifically

Vertebrate Animals:

Not Applicable (No Vertebrate Animals)

Biohazards:
Not Applicable (No Biohazards)

Resubmission:

- The application appears to be responsive to prior critique

Applications from Foreign Organizations:
Not Applicable (No Foreign Organizations)

Select Agents:
Not Applicable (No Select Agents)

Resource Sharing Plans:
Not Applicable (No Relevant Resources)

Authentication of Key Biological and/or Chemical Resources:
Not Applicable (No Relevant Resources)

Budget and Period of Support:
Recommend as Requested

THE FOLLOWING SECTIONS WERE PREPARED BY THE SCIENTIFIC REVIEW OFFICER TO SUMMARIZE THE OUTCOME OF DISCUSSIONS OF THE REVIEW COMMITTEE, OR REVIEWERS' WRITTEN CRITIQUES, ON THE FOLLOWING ISSUES:

PROTECTION OF HUMAN SUBJECTS: ACCEPTABLE

INCLUSION OF WOMEN PLAN: ACCEPTABLE

INCLUSION OF MINORITIES PLAN: ACCEPTABLE

INCLUSION ACROSS THE LIFESPAN: ACCEPTABLE

COMMITTEE BUDGET RECOMMENDATIONS: The budget was recommended as requested.

Footnotes for 1 R34 MH126776-01A1; PI Name: Mitchell, Jason W

+ Derived from the range of percentile values calculated for the study section that reviewed this application.

NIH has modified its policy regarding the receipt of resubmissions (amended applications).See Guide Notice NOT-OD-18-197 at https://grants.nih.gov/grants/guide/notice-files/NOT-OD-18-197.html. The impact/priority score is calculated after discussion of an application by averaging the overall scores (1-9) given by all voting reviewers on the committee and multiplying by 10. The criterion scores are submitted prior to the meeting by the individual reviewers assigned to an application, and are not discussed specifically at the review meeting or calculated into the overall impact score. Some applications also receive a percentile ranking. For details on the review process, see http://grants.nih.gov/grants/peer_review_process.htm#scoring.

MEETING ROSTER

HIV/AIDS Intra- and Inter-personal Determinants and Behavioral Interventions Study Section
Risk, Prevention and Health Behavior Integrated Review Group
CENTER FOR SCIENTIFIC REVIEW
HIBI
03/15/2021 - 03/16/2021

Notice of NIH Policy to All Applicants: Meeting rosters are provided for information purposes only. Applicant investigators and institutional officials must not communicate directly with study section members about an application before or after the review. Failure to observe this policy will create a serious breach of integrity in the peer review process, and may lead to actions outlined in NOT-OD-14-073 at https://grants.nih.gov/grants/guide/notice-files/NOT-OD-14-073.html and NOT-OD-15-106 at https://grants.nih.gov/grants/guide/notice-files/NOT-OD-15-106.html, including removal of the application from immediate review.

CHAIRPERSON(S)

VREEMAN, RACHEL CHRISTINE, MD
PROFESSOR
DEPARTMENT OF HEALTH SYSTEM DESIGN
AND GLOBAL HEALTH
ICAHN SCHOOL OF MEDICINE AT MOUNT SINAI
NEW YORK CITY, NY 10029

MEMBERS

ASSOUMOU, SABRINA A, MD, MPH *
ASSISTANT PROFESSOR
DEPARTMENT OF MEDICINE
BOSTON UNIVERSITY SCHOOL OF MEDICINE
BOSTON, MA 02118

BALAN, IVAN C, PHD *
ASSOCIATE PROFESSOR
CENTER FOR TRANSLATIONAL BEHAVIORAL SCIENCE
COLLEGE OF MEDICINE
FLORIDA STATE UNIVERSITY
TALLAHASSEE, FL 32310

BAZZI, ANGELA ROBERTSON, MPH, PHD *
ASSOCIATE PROFESSOR
HERBERT WERTHEIM SCHOOL OF PUBLIC HEALTH AND
HUMAN LONGEVITY SCIENCE
UNIVERSITY OF CALIFORNIA, SAN DIEGO
SAN DIEGO, CA 92093

BUTLER, LISA MICHELLE, PHD
ASSOCIATE RESEARCH PROFESSOR
INSTITUTE FOR COLLABORATION ON HEALTH,
INTERVENTION, AND POLICY
UNIVERSITY OF CONNECTICUT
STORRS, CT 06269

CHRISTOPOULOS, KATERINA A, MD, MPH *
ASSOCIATE PROFESSOR
HIV/AIDS DIVISION
SAN FRANCISCO GENERAL HOSPITAL
UNIVERSITY OF CALIFORNIA, SAN FRANCISCO
SAN FRANCISCO, CA 94110

COMULADA, WARREN SCOTT, DRPH
ASSOCIATE PROFESSOR
DEPARTMENT OF PSYCHIATRY
AND BIOBEHAVIORAL SCIENCES
SCHOOL OF PUBLIC HEALTH
UNIVERSITY OF CALIFORNIA, LOS ANGELES
LOS ANGELES, CA 90024

DODGE, BRIAN MARK, PHD *
PROFESSOR
DEPARTMENT OF APPLIED HEALTH SCIENCE CENTER
SEXUAL HEALTH PROMOTION
SCHOOL OF PUBLIC HEALTH
INDIANA UNIVERSITY
BLOOMINGTON, IN 47405

GAMAREL, KRISTINE E, PHD *
JOHN G. SEARLE ASSISTANT PROFESSOR
DEPARTMENT OF HEALTH BEHAVIOR
AND HEALTH EDUCATION
SCHOOL OF PUBLIC HEALTH
UNIVERSITY OF MICHIGAN
ANN ARBOR, MI 48109

GRAHAM, SUSAN MARIE, MD, PHD
ASSOCIATE PROFESSOR
DIVISION OF ALLERGY AND INFECTIOUS DISEASES
DEPARTMENTS OF MEDICINE AND GLOBAL HEALTH
SCHOOL OF MEDICINE
UNIVERSITY OF WASHINGTON
SEATTLE, WA 98104

GROV, CHRISTIAN, PHD
PROFESSOR AND CHAIR
DEPARTMENT OF COMMUNITY HEALTH
AND SOCIAL SCIENCES
SCHOOL OF PUBLIC HEALTH AND HEALTH POLICY
CITY UNIVERSITY OF NEW YORK
NEW YORK, NY 10027

HANSEN, NATHAN B, PHD
DEPARTMENT HEAD AND PROFESSOR
DEPARTMENT OF HEALTH PROMOTION AND BEHAVIOR
COLLEGE OF PUBLIC HEALTH
UNIVERSITY OF GEORGIA
ATHENS, GA 30602

HORVATH, KEITH JOSEPH, PHD
ASSOCIATE PROFESSOR
DEPARTMENT OF CLINICAL PSYCHOLOGY
SAN DIEGO STATE UNIVERSITY
SAN DIEGO, CA 92120

KIPKE, MICHELE D, PHD
PROFESSOR
DEPARTMENTS OF PEDIATRICS AND PREVENTIVE
MEDICINE
KECK SCHOOL OF MEDICINE
UNIVERSITY OF SOUTHERN CALIFORNIA
LOS ANGELES, CA 90027

KIPP, AARON M, PHD *
ASSISTANT PROFESSOR
DEPARTMENT OF PUBLIC HEALTH
EAST CAROLINA UNIVERSITY BRODY SCHOOL OF MEDICINE
GREENVILLE, NC 27858

LEGRAND, SARA H, PHD *
ASSOCIATE RESEARCH PROFESSOR
DUKE GLOBAL HEALTH INSTITUTE
DUKE UNIVERSITY
DURHAM , NC 27710

LEVY, JUDITH A, PHD *
ASSOCIATE PROFESSOR
THE HEALTH POLICY AND ADMINISTRATION DIVISION
SCHOOL OF PUBLIC HEALTH
UNIVERSITY OF ILLINOIS AT CHICAGO
CHICAGO, IL 60612

LUSENO, WINFRED K, PHD *
SENIOR RESEARCH SCIENTIST
PACIFIC INSTITUTE FOR RESEARCH AND EVALUATION
CHAPEL HILL, NC 27514

PARCESEPE, ANGELA, MPH, PHD *
ASSISTANT PROFESSOR
DEPARTMENT OF MATERNAL AND CHILD HEALTH
GILLINGS SCHOOL OF GLOBAL PUBLIC HEALTH
UNIVERSITY OF NORTH CAROLINA AT CHAPEL HILL
CHAPEL HILL, NC 27599

RAMSEY, SUSAN E, PHD
ASSOCIATE PROFESSOR
DIVISION OF GENERAL INTERNAL MEDICINE
RHODE ISLAND HOSPITAL
BROWN UNIVERSITY
PROVIDENCE, RI 02903

SSEWAMALA, FRED M, PHD
PROFESSOR
INSTITUTE FOR PUBLIC HEALTH
BROWN SCHOOL
WASHINGTON UNIVERSITY
ST. LOUIS, MO 63130

STOCKMAN, JAMILA KINSHASA, PHD
ASSOCIATE PROFESSOR
DIVISION OF GLOBAL PUBLIC HEALTH
DEPARTMENT OF MEDICINE
SCHOOL OF MEDICINE
UNIVERSITY OF CALIFORNIA, SAN DIEGO
LA JOLLA, CA 92093

SULLIVAN, PATRICK SEAN, PHD
PROFESSOR
DEPARTMENT OF EPIDEMIOLOGY
ROLLINS SCHOOL OF PUBLIC HEALTH
EMORY UNIVERSITY
ATLANTA, GA 30322

THAMES, APRIL D, PHD
ASSOCIATE PROFESSOR
DEPARTMENT OF PSYCHOLOGY
UNIVERSITY OF SOUTHERN CALIFORNIA
LOS ANGELES, CA 90089

TIEU, HONG VAN NHU, MD *
HEAD
LABORATORY OF INFECTIOUS DISEASE PREVENTION
LINDSLEY F KIMBALL RESEARCH INSTITUTE
NEW YORK BLOOD CENTER
NEW YORK, NY 10065

TOBIN, KARIN E, PHD
ASSOCIATE PROFESSOR
DEPARTMENT OF HEALTH, BEHAVIOR, AND SOCIETY
BLOOMBERG SCHOOL OF PUBLIC HEALTH
JOHNS HOPKINS UNIVERSITY
BALTIMORE, MD 21205

TURAN, JANET M, PHD
PROFESSOR
DEPARTMENT OF HEALTH CARE ORGANIZATION
AND POLICY
SCHOOL OF PUBLIC HEALTH
UNIVERSITY OF ALABAMA AT BIRMINGHAM
BIRMINGHAM, AL 35294

WEBEL, ALLISON R, PHD
ASSOCIATE PROFESSOR
FRANCIS PAYNE BOLTON SCHOOL OF NURSING
CASE WESTERN RESERVE UNIVERSITY
CLEVELAND, OH 44106

WINDSOR, LILIANE CAMBRAIA, PHD
ASSOCIATE PROFESSOR
SCHOOL OF SOCIAL WORK
THE UNIVERSITY OF ILLINOIS AT URBANA-CHAMPAIGN
URBANA, IL 61801

MAIL REVIEWER(S)

CAMP, CAMERON J, PHD
DIRECTOR OF RESEARCH AND DEVELOPMENT
CENTER FOR APPLIED RESEARCH IN DEMENTIA
SALON, OH 44139

SCIENTIFIC REVIEW OFFICER

RUBERT, MARK P, PHD
SCIENTIFIC REVIEW OFFICER
CENTER FOR SCIENTIFIC REVIEW
NATIONAL INSTITUTES OF HEALTH
BETHESDA, MD 20892

EXTRAMURAL SUPPORT ASSISTANT

CAMBRELEN, AMY ANGELA
EXTRAMURAL SUPPORT ASSISTANT
CENTER FOR SCIENTIFIC REVIEW
NATIONAL INSTITUTE OF HEALTH
BETHESDA, MD 20892

* Temporary Member. For grant applications, temporary members may participate in the entire meeting or may review only selected applications as needed.

Consultants are required to absent themselves from the room during the review of any application if their presence would constitute or appear to constitute a conflict of interest.
